# A prognostic nutritional index-based nomogram for predicting postoperative survival in stages I–III rectal cancer patients

**DOI:** 10.3389/fnut.2025.1680287

**Published:** 2025-09-11

**Authors:** Ling Liu, Chu Lei, Li Li, Xi Peng, Haiyan Gong, Anhong Hu

**Affiliations:** ^1^Department of Prevention & Healthcare, The First Affiliated Hospital, Army Medical University, Chongqing, China; ^2^Department of Information, The First Affiliated Hospital, Army Medical University, Chongqing, China

**Keywords:** rectal cancer, prognostic nutritional index, prediction model, survival, nomogram

## Abstract

**Introduction:**

Rectal cancer (RC) is a common malignancy of the digestive system with both high incidence and mortality. Its prognosis is influenced by multiple factors, with nutritional status playing a pivotal role. However, current prognostic models rarely incorporate this factor.

**Methods:**

To address this gap, we have developed a novel prognostic nomogram. The newly constructed Prognostic Nutritional Index (PNI)-incorporated nomogram incorporates preoperative pathological tumor-node-metastasis (pTNM) stage, preoperative PNI, preoperative serum carcinoembryonic antigen (CEA) levels, intraoperative blood loss (IBL), and postoperative serum CEA levels.

**Results:**

Our analysis showed that preoperative PNI ≤ 47.15, preoperative CEA > 14.13 ng/mL, IBL > 130 mL, postoperative CEA > 4.8 ng/mL, and advanced pTNM stage were independent risk factors for poor survival in patients with stage I-III rectal cancer. Compared with the non-PNI nomograms (combining preoperative CEA, postoperative CEA, pTNM and IBL, but without PNI) and the conventional pTNM staging models, the C-index of the PNI-incorporated nomogram is 0.721, compared to 0.710 for non-PNI nomograms and 0.636 for pTNM staging models, demonstrating improved predictive performance. Furthermore, the PNI-incorporated nomogram achieved AUC values of 0.855, 0.759, and 0.717 for 1, 3, and 5 year overall survival prediction, respectively, in the training set, and 0.952, 0.682, and 0.658 for the corresponding time points in the validation set.

**Conclusion:**

This model significantly improves existing prognostic methods and provides clinicians with a more comprehensive and clinically applicable tool for predicting outcomes in patients with RC.

## 1 Introduction

Colorectal cancer (CRC), including Rectal cancer (RC) and colon cancer (CC), is one of the most common malignancies in the digestive system and ranks third in global cancer incidence. It was estimated that more than 1.9 million new CRC cases and approximately 0.93 million related deaths worldwide in 2020 (1). In recent years, the incidence and mortality of CRC have been rising continuously. According to the 2020 China Cancer Statistics Report, the incidence and mortality of CRC ranked 2nd and 5th in China (2). Among them, the incidence of RC in China is similar to that of CC, with an increasing proportion of cases occurring in younger individuals, accounting for about 10–15% of all CRC cases. The standard treatment for CRC is a combination of surgery, chemotherapy, and radiation therapy (3). Currently, the 1-, 3-, and 5-year survival rates of CRC patients in China are 0.79, 0.72, and 0.62, respectively (4). In addition to the established prognostic factors, such as pathological tumor-node-metastasis (pTNM) stage, carcinoembryonic antigen (CEA) levels, and treatment strategies, increasing attention has been directed toward additional variables that may refine the accuracy of postoperative prognosis assessment in RC patients.

Previous studies have demonstrated that preoperative serum CEA levels and tumor histological grade are significant determinants of patient prognosis (5, 6). Additionally, intraoperative blood loss (IBL) has been identified as a potential risk factor for postoperative peritoneal recurrence in stage CRC patients, adversely affecting survival (7). The prognostic nutritional index (PNI), calculated from serum albumin levels and peripheral lymphocyte count, serves as a critical indicator of both nutritional and inflammatory status. Originally proposed by Onodera et al. in 1984 for surgical risk assessment (8), PNI has increasingly been applied to evaluate survival outcomes in various malignancies (9, 10). In esophageal cancer, PNI has been established as an independent prognostic factor, reflecting the patient’s nutritional and immune status, which in turn influences tumor progression, metastasis, and clinical outcomes (11, 12). Across multiple cancer types, a low PNI correlates with poor prognosis, including reduced overall survival (OS), disease-free survival (DFS), and progression-free survival (PFS) (13). Specifically in CRC, patients with low PNI exhibit significantly worse OS and DFS compared to those with high PNI (14). Preoperative nutritional status, as indicated by PNI, may thus be closely associated with prognosis in patients undergoing curative resection for CRC (15). Despite these findings, consensus is still lacking, and no standardized tools currently exist to integrate these readily available indicators into precise prognostic models for RC.

In the present study, we systematically evaluated the prognostic impact of tumor-related laboratory markers, PNI, IBL, and other indicators on postoperative survival in stage I-III RC patients, and identified independent risk factors associated with survival. Based on these risk factors, a new simple and reliable scoring system for the survival rate of postoperative RC patients was developed (PNI-incorporated nomogram: combining preoperative PNI, preoperative CEA, postoperative CEA, pTNM, and IBL), which can evaluate the survival of patients.

## 2 Materials and methods

### 2.1 Research ethics committee approval

This study was approved by the Medical Ethics Committee of The First Affiliated Hospital of Army Medical University (No.(B)KY2025019) and was conducted in accordance with the Declaration of Helsinki.

### 2.2 Patients

**Figure 1:**
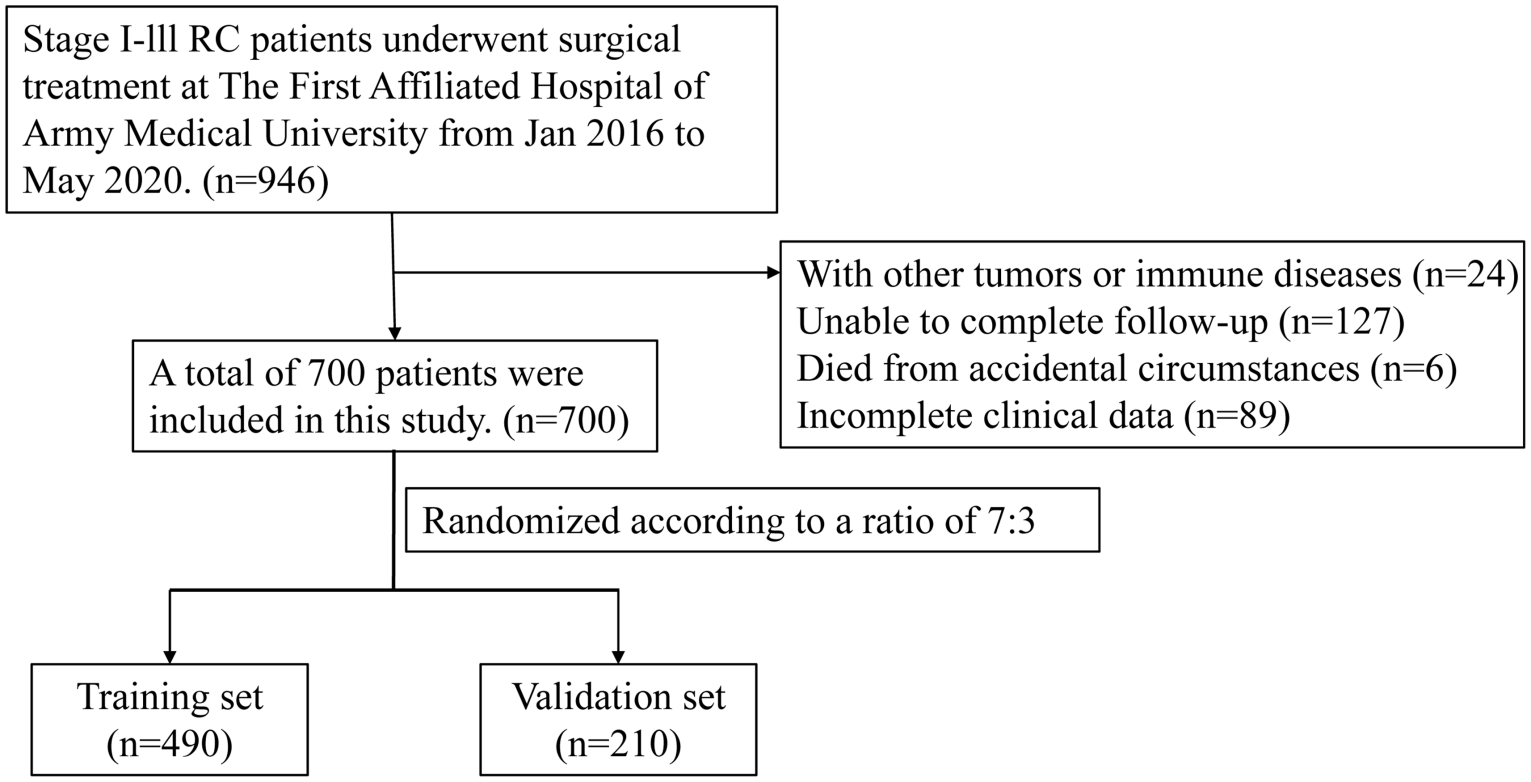
The flow diagram of stages I–III RC patients underwent surgical treatment enrolled in this study.

We retrospectively analyzed patients with stage I–III RC who were diagnosed and underwent surgical treatment at The First Affiliated Hospital of Army Medical University between January 2016 and May 2020. All patients were pathologically staged using the internationally recognized pTNM staging system, which was established by the American Joint Committee on Cancer (AJCC). Among them, patients with high-risk factors received postoperative adjuvant therapy with chemotherapy or chemoradiotherapy. The inclusion criteria were as follows: (1) patients diagnosed with RC pathologically from January 2016 to May 2020; (2) pTNM stage I-III; (3) patients who underwent surgical treatment; (4) patients who did not receive nutritional support before surgery. (5) age > 18 years. The exclusion criteria were as follows: (1) patients with other malignant tumors or immune diseases; (2) patients who were unable to complete follow-up; (3) patients who died due to accidental circumstances; (4) patients with incomplete clinical data. Overall, a total of 946 patients with stage I-III RC who underwent surgical treatment were initially collected. Based on the inclusion and exclusion criteria, 700 patients were ultimately enrolled in this study. These patients were randomly allocated into a training set (*n* = 490) and a validation set (*n* = 210) at a ratio of 7:3 (Figure 1). Relevant demographic information and clinical data were obtained by reviewing electronic medical records.

### 2.3 Clinical and laboratory information

Data on patient demographics (including age, sex, and body mass index), clinical characteristics, and laboratory parameters were collected. These laboratory parameters included white blood cell count (WBC), neutrophil count, lymphocyte count, monocyte count, platelet count, hemoglobin, CEA, serum albumin, globulin, total protein, neutrophil-to-lymphocyte ratio (NLR), platelet-to-lymphocyte ratio (PLR), systemic immune-inflammation index (SII), and PNI. Measurements were taken within 1 week preoperatively and at 6 months postoperatively. SII was calculated using the formula: Platelet count × Neutrophil count/Lymphocyte count, and PNI was calculated using the formula: Serum albumin (g/L) + 5 × Lymphocyte count (×10^9^/L). The optimal cut-off values for these indicators were determined using X-tile software version 3.6.1 (Yale University, New Haven, CT, United States).

### 2.4 Postoperative data collection

All patient data collected in this study were obtained exclusively from the hospital’s electronic medical record database. These data include follow-up records performed every 3 months for the first 3 years and every 6 months for 3 to 5 years postoperatively. The follow-up data included the patient’s general health status, blood indicators (complete blood count, liver and kidney function, tumor markers, etc.), and imaging examinations. The cutoff time for follow-up data collection was May 2025. OS is defined as the time interval from the date of surgery to the date of last follow-up (date of death or date of end of follow-up). In this study, 3- and 5-year OS was used as the criterion for evaluating patient prognosis.

### 2.5 Statistical analysis

SPSS 21.0 and R-based MedCalc (version 19.5.6) software were used for data analysis. The continuous variables with non-normal distribution were expressed as *M*(P25, P75), and the Mann–Whitney *U* test was used to analyze the comparison between groups. Categorical variables are expressed as percentages and compared between groups using the chi-square (*χ*^2^) test. The Cox proportional hazards regression model was applied to identify independent risk factors affecting postoperative survival rates through univariate and multivariate analyses. A nomogram was constructed using R software with the rms package, and its performance characteristics were examined using calibration plots. The predictive performance of the nomogram model was evaluated by Harrell’s concordance index (C-index), receiver operating characteristic (ROC) curve, decision curve analysis (DCA), and time-dependent ROC curve analysis. The predictive abilities of the PNI-incorporated nomogram (Model A), the non-PNI nomogram (combining preoperative CEA, postoperative CEA, pTNM and IBL, but without PNI, Model B) and pTNM staging model (Model C) were assessed through decision curve analysis. *p*-value <0.05 was considered statistically significant.

## 3 Results

### 3.1 Baseline demographic characteristics

The median follow-up time was 76.5 months (range: 5–112 months) in the training set and 77 months (range: 6–112 months) in the validation set. The 3-year and 5-year OS rates were 83.7 and 74.3% in the training set, and 86.2 and 74.3% in the validation set, respectively. As shown in Table 1, no statistically significant differences were observed in the clinical characteristics of enrolled patients between the training and validation sets (*p* > 0.05). More detailed baseline demographics and disease characteristics can be found in Supplementary Table S1.

**Table 1:** Clinical characteristics of patients in the training set and validation set.

Characteristics	Training set *N* = 490	Validation set *N* = 210	*p*-value
Age (year)	57.0[50.0;65.0]	60.0[51.0;66.0]	0.173
Age (year)			0.233
≤60	273(55.7%)	106(50.5%)	
>60	217(44.3%)	104(49.5%)	
Sex			0.959
Female	177(36.1%)	77(36.7%)	
Male	313(63.9%)	133(63.3%)	
BMI			0.356
≤20.08	69(14.1%)	36(17.1%)	
>20.08	421(85.9%)	174(82.9%)	
Grade			0.232
Well/moderately differentiated	431(88.0%)	177(84.3%)	
Poorly differentiated/undifferentiated	59(12.0%)	33(15.7%)	
Classification			1.000
Protruded type	188(38.4%)	80(38.1%)	
Ulcerative/infiltrative type	302(61.6%)	130(61.9%)	
pTNM staging			0.788
I–II	294(60.0%)	123(58.6%)	
III	196(40.0%)	87(41.4%)	
Preoperative WBC (×10^9^/L)			0.307
≤4.46	59(12.0%)	19(9.05%)	
>4.46	431(88.0%)	191(91.0%)	
Preoperative Monocyte (×10^9^/L)			0.696
≤0.30	189(38.6%)	77(36.7%)	
>0.30	301(61.4%)	133(63.3%)	
Preoperative Neutrophil (×10^9^/L)			0.474
≤2.48	50(10.2%)	26(12.4%)	
>2.48	440(89.8%)	184(87.6%)	
Preoperative eosinophil (×10^9^/L)			0.382
≤0.11	249(50.8%)	115(54.8%)	
>0.11	241(49.2%)	95(45.2%)	
Preoperative PLT (×10^9^/L)			0.721
≤145	60(12.2%)	23(11.0%)	
>145	430(87.8%)	187(89.0%)	
Preoperative PNI			1.000
≤47.15	106(21.6%)	46(21.9%)	
>47.15	384(78.4%)	164(78.1%)	
Preoperative globulin (g/L)			0.594
≤33.90	440(89.8%)	185(88.1%)	
>33.90	50(10.2%)	25(11.9%)	
Preoperative CEA (ng/mL)			0.254
≤14.13	432(88.2%)	192(91.4%)	
>14.13	58(11.8%)	18(8.57%)	
Operating_time (min)			0.817
≤140	75(15.3%)	30(14.3%)	
>140	415(84.7%)	180(85.7%)	
IBL (ml)			0.397
≤130	224(45.7%)	88(41.9%)	
>130	266(54.3%)	122(58.1%)	
Postoperative WBC (×10^9^/L)			0.636
≤4.03	64(13.1%)	24(11.4%)	
>4.03	426(86.9%)	186(88.6%)	
Postoperative basophil (×10^9^/L)			0.538
≤0.02	280(57.1%)	114(54.3%)	
>0.02	210(42.9%)	96(45.7%)	
Postoperative SII			0.275
≤665.80	419(85.5%)	172(81.9%)	
>665.80	71(14.5%)	38(18.1%)	
Postoperative NLR			0.286
≤4.22	442(90.2%)	183(87.1%)	
>4.22	48(9.80%)	27(12.9%)	
Postoperative PLR			0.327
≤185.14	441(90.0%)	183(87.1%)	
>185.14	49(10.0%)	27(12.9%)	
Postoperative PNI			0.541
≤45.80	88(18.0%)	33(15.7%)	
>45.80	402(82.0%)	177(84.3%)	
Postoperative CEA (ng/mL)			0.980
≤4.80	432(88.2%)	186(88.6%)	
>4.80	58(11.8%)	24(11.4%)	

BMI, body mass index; pTNM, pathological tumor-node-metastasis; WBC, white blood cell; PLT, platelets; PNI, Prognostic Nutritional Index; CEA, carcinoembryonic antigen; IBL, intraoperative blood loss; SII, systemic immune-inflammation index; NLR, neutrophil-to-lymphocyte ratio; PLR, platelet-to-lymphocyte ratio. *P* < 0.05 means statistically significant.

### 3.2 Univariate and multivariate cox regression results

As shown in Tables 2, 3, univariate and multivariate Cox regression analyses indicated that preoperative PNI [hazard ratio (HR) 0.551, 95% CI 0.370–0.819, *p* = 0.003], preoperative CEA (HR 1.907, 95% CI 1.220–2.980, *p* = 0.005), pTNM stage (HR 1.636, 95% CI 1.147–2.333, *p* = 0.007), IBL (HR 1.769, 95% CI 1.215–2.576, *p* = 0.003), and postoperative CEA (HR 4.240, 95% CI 2.805–6.408, *p* < 0.001) were identified as independent influencing factors for postoperative survival in RC patients (*p* < 0.05). Although tumor grade and classification also reached statistical significance in multivariate Cox analysis (Table 3), further model comparison showed that adding these two variables did not improve model discrimination or calibration. Variance inflation factor (VIF) analysis showed no evidence of multicollinearity among the variables selected by multivariate Cox regression analyses (all VIFs < 2; Supplementary Table S2). Therefore, to maintain the parsimony and stability of the model, we adopted a more stringent screening threshold of *p* < 0.01, which resulted in these indicators not being included in the final PNI-incorporated nomogram.

**Table 2:** Univariate analysis of OS of RC patients undergoing surgical treatment in the training set.

Variable	HR	Lower_95CI	Upper_95CI	*p*_value
Sex	1.304	0.898	1.893	0.164
Age, median	1.009	0.992	1.026	0.313
Age (≤60, >60)	1.408	0.997	1.990	0.052
BMI	0.681	0.434	1.070	0.096
Preoperative WBC	0.610	0.386	0.965	0.035
Preoperative monocyte	0.695	0.492	0.983	0.040
Preoperative neutrophil	0.588	0.361	0.957	0.033
Preoperative eosinophil	0.657	0.462	0.935	0.019
Preoperative PLT	0.698	0.437	1.114	0.132
Preoperative PNI	0.557	0.383	0.812	0.002
Preoperative globulin	0.719	0.377	1.371	0.316
Preoperative CEA	2.769	1.830	4.190	<0.001
Operating time	2.146	1.157	3.980	0.015
IBL	1.898	1.313	2.744	0.001
Grade	2.149	1.388	3.326	0.001
Classification	2.046	1.372	3.052	<0.001
pTNM staging	1.721	1.219	2.431	0.002
Postoperative WBC	0.755	0.473	1.206	0.240
Postoperative basophil	1.378	0.975	1.945	0.069
Postoperative SII	2.371	1.589	3.536	<0.001
Postoperative NLR	2.268	1.433	3.590	<0.001
Postoperative PLR	2.487	1.584	3.906	<0.001
Postoperative PNI	0.773	0.506	1.182	0.235
Postoperative CEA	5.386	3.687	7.869	<0.001

HR, hazard ratio; BMI, body mass index; WBC, white blood cell; PLT, platelets; PNI, Prognostic Nutritional Index; CEA, carcinoembryonic antigen; IBL, intraoperative blood loss; pTNM, pathological tumor-node-metastasis; SII, systemic immune-inflammation index; NLR, neutrophil-to-lymphocyte ratio; PLR, platelet-to-lymphocyte ratio. *P* < 0.05 means statistically significant.

**Table 3:** Multivariate analysis of OS of RC patients undergoing surgical treatment in the training set.

Variable	HR	Lower_95CI	Upper_95CI	*p*_value
Preoperative WBC	0.830	0.396	1.735	0.620
Preoperative monocyte	0.714	0.490	1.043	0.081
Preoperative neutrophil	0.658	0.307	1.410	0.281
Preoperative eosinophil	0.722	0.499	1.045	0.084
Preoperative PNI	0.551	0.370	0.819	0.003*
Preoperative CEA	1.907	1.220	2.980	0.005*
Operating time	1.668	0.894	3.114	0.108
IBL	1.769	1.215	2.576	0.003*
Grade	1.801	1.141	2.843	0.011
Classification	1.707	1.134	2.568	0.010
pTNM staging	1.636	1.147	2.333	0.007*
Postoperative SII	2.288	1.162	4.505	0.017
Postoperative NLR	1.021	0.531	1.960	0.951
Postoperative PLR	1.039	0.536	2.017	0.909
Postoperative CEA	4.240	2.805	6.408	<0.001*

HR, hazard ratio; WBC, white blood cell; PNI, Prognostic Nutritional Index; CEA, carcinoembryonic antigen; IBL, intraoperative blood loss; pTNM, pathological tumor-node-metastasis; SII, systemic immune-inflammation index; NLR, neutrophil-to-lymphocyte ratio; PLR, platelet-to-lymphocyte ratio. *indicates variables that met the inclusion criteria for the model (*p* < 0.01).

### 3.3 Construction of the nomogram

**Figure 2:**
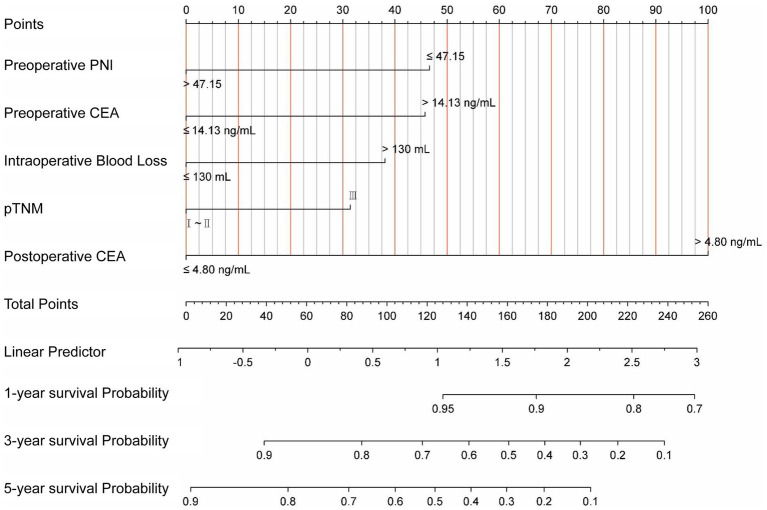
Nomograms used to predict 1-, 3-, and 5-year OS of RC patients treated by surgery. PNI, prognostic nutrition index; CEA, carcinoembryonic antigen; pTNM, pathological tumor-node-metastasis.

Based on the results of univariate and multivariate Cox regression, a survival prediction nomogram for RC patients was constructed using the following five independent prognostic factors: preoperative PNI, preoperative CEA, pTNM staging, IBL, and postoperative CEA (Figure 2).

### 3.4 Validation of the nomogram

**Figure 3:**
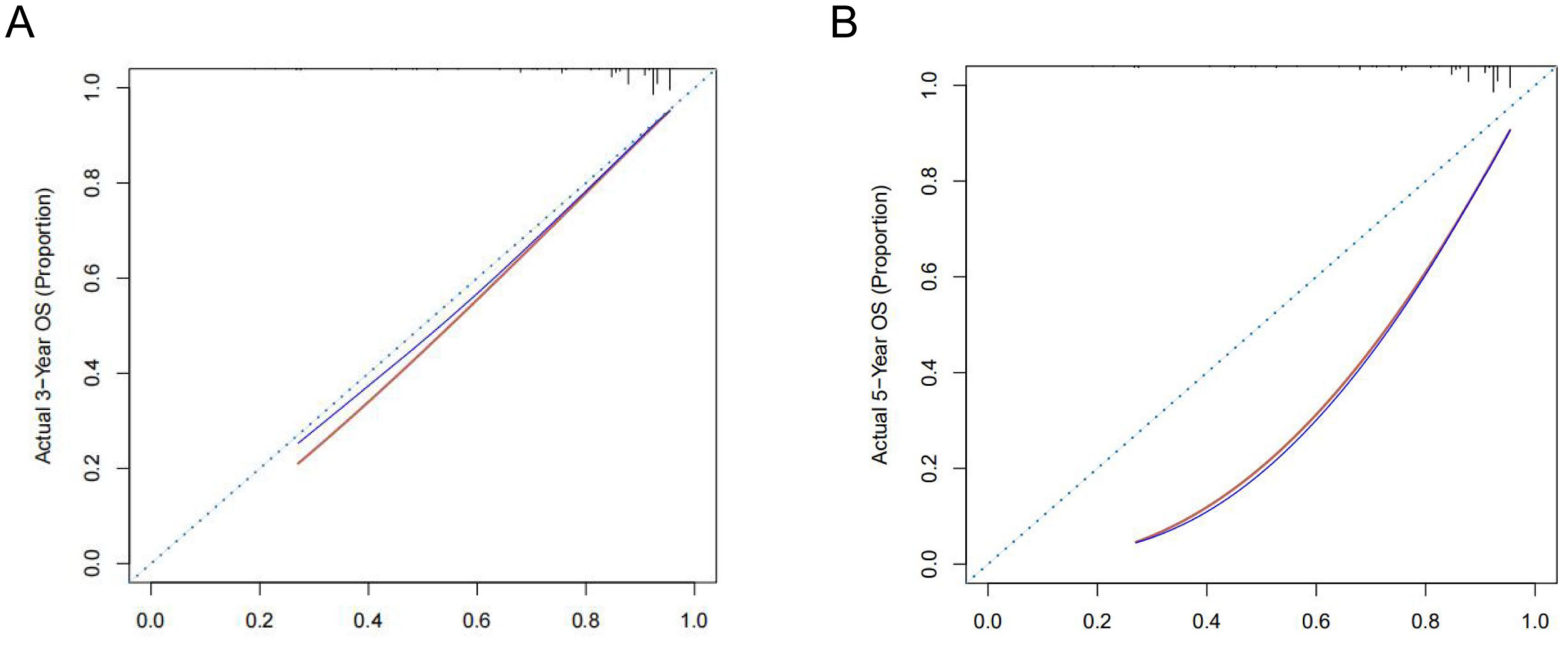
Calibration curves for 3- **(A)** and 5-year **(B)** OS of rectal cancer patients with surgical treatment.

**Figure 4:**
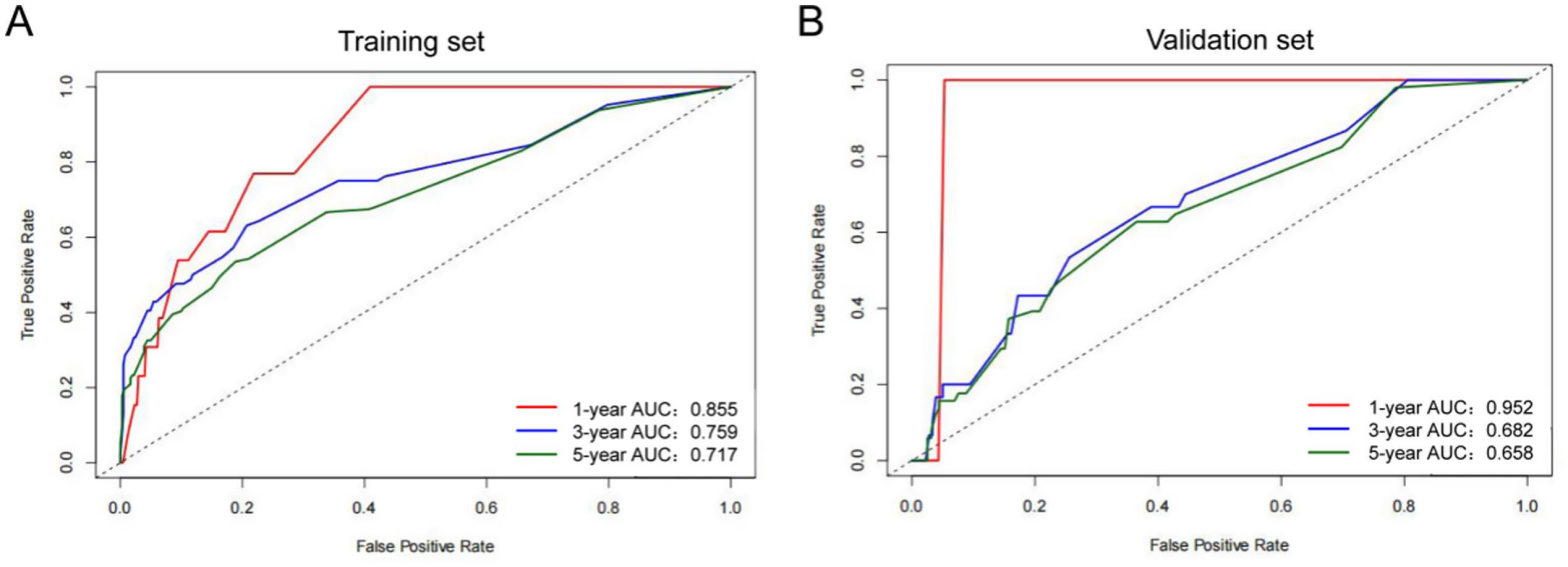
Receiver operating characteristic curve for OS of RC patients undergoing surgical treatment based on the nomogram. **(A)** ROC curve for 1-, 3-, and 5-year OS based on the nomogram in the training set. **(B)** ROC curve for 1-, 3-, and 5-year OS based on the nomogram in the validation set. AUC, area under the curve.

Further validation of the PNI-incorporated nomogram showed that the calibration curve (Figures 3A,B) was very close to the ideal curve, indicating strong agreement between the nomogram prediction rate and the actual postoperative survival rate of RC patients, demonstrating high predictive accuracy. The PNI-incorporated nomogram provided precise predictions for 3- and 5-year OS, and the prediction of 3-year OS is more reliable than that of 5-year OS. ROC curve analysis showed that the AUC values for 1-, 3-, and 5-year OS predictions were 0.855, 0.759, and 0.717 (Figure 4A), while the AUC values in the validation set were 0.952, 0.682, and 0.658 (Figure 4B). The results showed that the PNI-incorporated nomogram could accurately predict the postoperative survival of RC patients.

**Figure 5:**
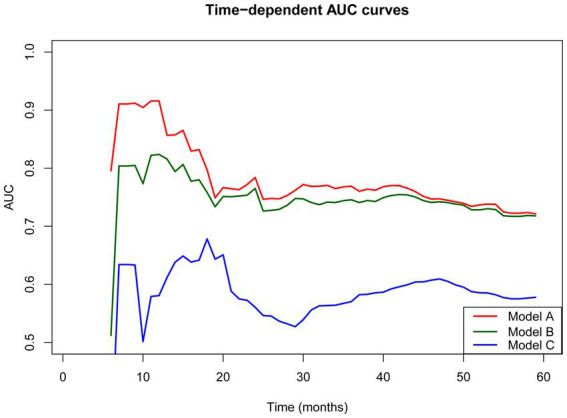
Time-dependent receiver-operating characteristic curves for the PNI-incorporated nomogram (Model A), the non PNI-nomogram model (Model B) and pTNM staging model (Model C) for the prediction of overall survival. AUC, area under the curve; PNI, prognostic nutrition index; pTNM, pathological tumor-node-metastasis.

In addition, the prediction consistency of the three prognostic models (Figure 5) was comparatively analyzed, and the results showed that the PNI-incorporated nomogram (Model A) was better than the non-PNI nomogram model (Model B) and the pTNM staging model (Model C). Specifically, the C indexes of the three models were 0.721 (95%CI 0.672–0.771), 0.710 (95%CI 0.656–0.756) and 0.636 (95%CI 0.556–0.716), indicating that Model A provided the highest prediction accuracy in the evaluation model.

### 3.5 Clinical utility of the nomogram

**Figure 6:**
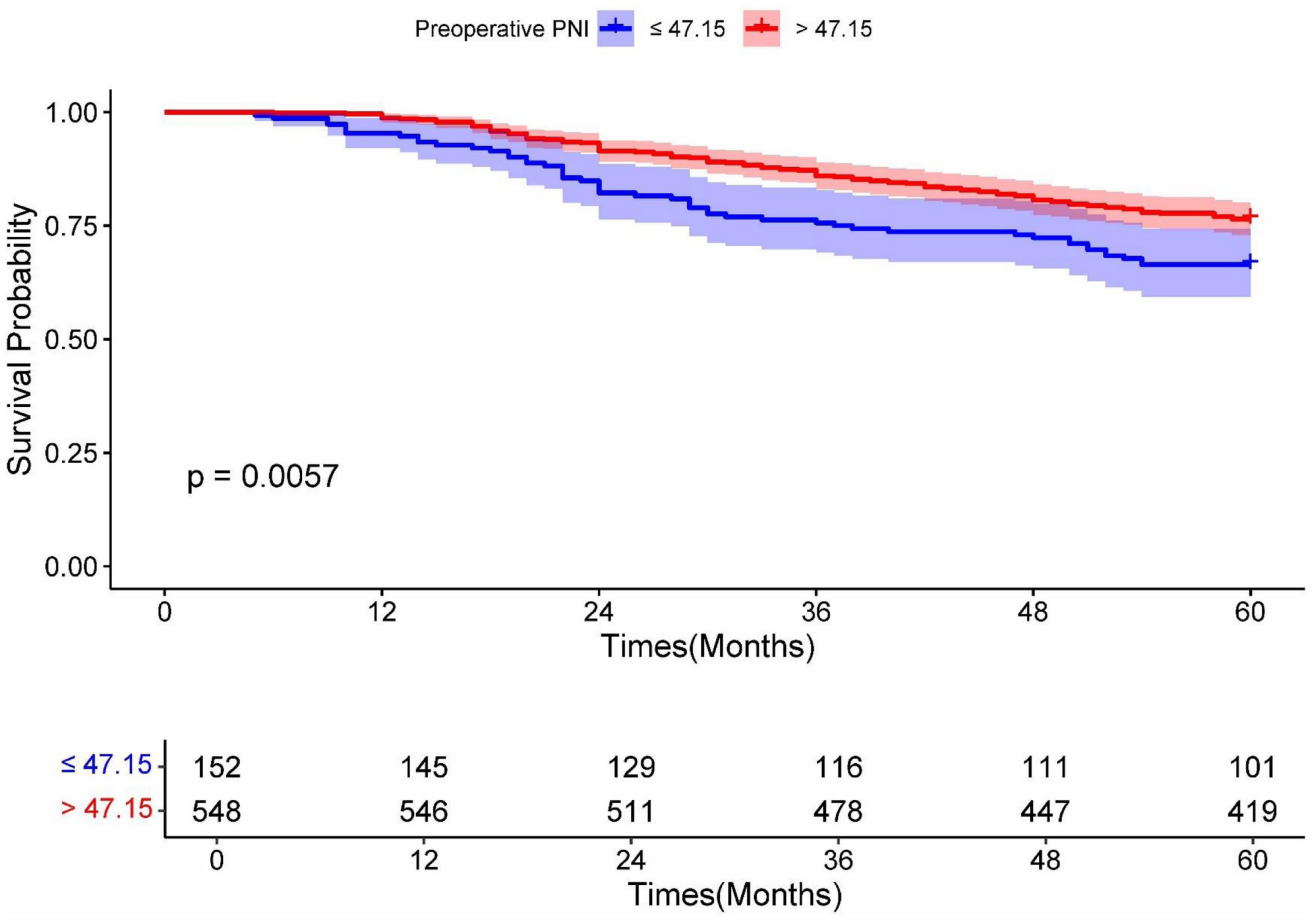
Kaplan–Meier analysis for OS of RC patients undergoing surgical treatment according to the preoperative PNI. Blue and red solid lines represent Kaplan–Meier analysis for OS according to preoperative PNI ≤ 47.15 and PNI > 47.15. PNI, prognostic nutrition index.

**Figure 7:**
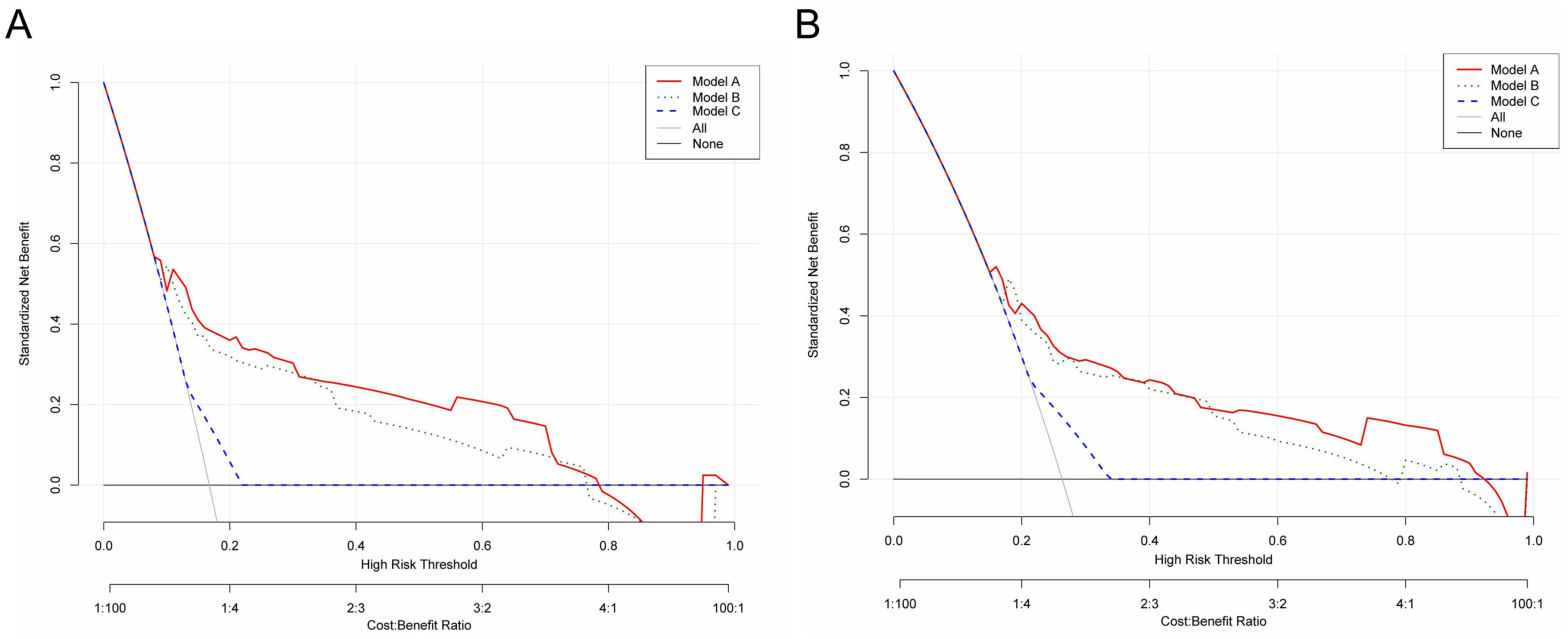
Decision curve analysis of 3- **(A)** and 5-year **(B)** OS predictions in RC patients who underwent surgical treatment in the training set. Model A, PNI-incorporated nomogram; Model B, the non PNI-nomogram model; Model C,pTNM staging model; PNI, prognostic nutrition index; pTNM, pathological tumor-node-metastasis.

Kaplan–Meier survival analysis revealed statistically significant differences in survival curves between the two groups (*p* = 0.0057), demonstrating that patients with preoperative PNI > 47.15 had more favorable survival outcomes (Figure 6). Furthermore, DCA showed superior clinical utility of the PNI-incorporated nomogram (Model A) compared to both the non-PNI-nomogram (Model B) and the pTNM staging model (Model C) across 3-year analyses (Figure 7A). Notably, Model A provided a greater net benefit in predicting 3-year survival than 5-year survival, particularly within the threshold range of 0.10 to 0.70 (Figure 7).

### 3.6 Online model visualization

The online version of our PNI-incorporated nomogram is publicly available at https://nctb.shinyapps.io/NCTB_model/. We anticipate this tool will assist clinicians and researchers in clinical decision-making. After entering a patient’s clinical characteristics, users can conveniently obtain time-dependent survival probability predictions through the automatically generated results and tables provided by the web server.

## 4 Discussion

The pTNM staging system is generally regarded as the gold standard for evaluating tumor prognosis. Currently, the pTNM stage serves as the primary indicator for assessing the prognosis of RC patients, thereby guiding clinicians in evaluating patients conditions and selecting postoperative treatment strategies. The pTNM staging mainly focuses on three preoperative indicators: tumor size (T), lymph node involvement (N), and distant metastasis (M), while it overlooks patient-specific factors such as physiological status (16). Clinical data showed significant prognostic heterogeneity among patients classified under the same pTNM stage who receives similar treatments. This discrepancy arises from differences in nutritional status, intraoperative conditions, and levels of circulating tumor marker. A study demonstrates shows that malnutrition is a practical indicator of various adverse outcomes following RC surgery (17). IBL also significantly affects survival outcomes. Elevated IBL levels have been associated with poorer long-term survival in CRC patients, regardless of blood transfusion (18). These findings collectively highlight the necessity of developing a comprehensive prognostic model that integrates individual patient characteristics to guide personalized therapeutic strategies for RC patients.

In recent years, there has been growing interest in the relationship between nutrition-related metabolic indicators and diseases. One study found that the Atherogenic Index of Plasma (AIP), calculated as log (triglycerides/HDL-C), demonstrates high sensitivity in diagnosing severe hepatic steatosis (19). Patients with metabolic dysfunction-associated steatotic liver disease (MASLD) often exhibit hyperhomocysteinemia (HHcy), which is associated with reduced vitamin D levels and alterations in glucose and lipid profiles (20). Among cancer patients, the prevalence of severe malnutrition is 19.3% (95% CI: 14.1–25.9%), representing a major factor contributing to adverse clinical outcomes and poor prognosis in this population (21). Furthermore, over 20% of cancer-related deaths are attributable to malnutrition (22). The PNI is calculated from serum albumin levels and peripheral lymphocyte counts, which is a comprehensive indicator of the patient’s nutritional and immunological status. Clinically, hypoalbuminemia impairs tissue repair capacity, reduces surgical tolerance, prolongs hospital stay, and adversely affects survival rates. Lymphopenia reflects impaired cellular immunity and reduces the tumoricidal effect of T cells and NK cells, thereby affecting the prognosis and survival of cancer patients (23). An increasing body of evidence confirms that PNI is an independent prognostic predictor and a validated biomarker for various malignancies (24, 25). A decreased PNI is often indicative of concurrent malnutrition and immunosuppression, which impairs the immune system’s capacity to monitor residual tumor cells and increases the risk of recurrence and distant metastasis (26). A meta-analysis showed that a low PNI is associated with increased levels of regulatory T cells and myeloid-derived suppressor cells in the tumor microenvironment, thereby promoting immune evasion. These findings suggest that the PNI may serve as a promising biomarker for predicting poor responses to immune checkpoint inhibitors in advanced cancers (27). Our study found that RC patients with a preoperative PNI > 47.15 undergoing surgery had a significant survival advantage. Helen Xie et al. (28) demonstrated that PNI is an independent risk factor for postoperative complications, PFS, and OS in RC patients, and that it is closely associated with postoperative survival outcomes. Consequently, PNI serves as a valuable complement to the pTNM staging system, a finding that is consistent with our own results.

The CEA is a glycoprotein serving as a broad-spectrum tumor marker, which is widely utilized for auxiliary diagnosis and prognostic evaluation across various malignancies (29–31). The normalization of postoperative tumor markers in patients with preoperative abnormalities has been associated with improved survival outcomes (32). A study of non-metastatic gastric cancer patients undergoing radical gastrectomy revealed that postoperative increases in CEA/CA19-9 levels served as superior prognostic indicators compared to preoperative values, with OS inversely correlating with the magnitude of postoperative tumor marker elevation (33). In CRC patients, preoperative CEA level is an important indicator for predicting recurrence and survival, and an elevation in postoperative CEA level is associated with adverse clinical features, including intestinal obstruction, perforation, advanced tumor stage, and the presence of lymphatic, vascular, or perineural invasion (34). Studies have revealed the predictive value of circulating tumor cell (CTC) count and serum CEA mRNA level in postoperative recurrence of digestive tract tumors, and the combination of the two can improve the diagnostic efficiency in predicting postoperative recurrence (35). In our study, both preoperative and postoperative CEA levels were identified as independent risk factors for predicting the survival of patients with stage I-III RC. Patients with a preoperative CEA level ≤14.13 ng/mL and a CEA level ≤4.80 ng/mL at 6 months after surgery had a significant survival advantage. Therefore, postoperative monitoring of CEA levels is also of great significance for the prognostic evaluation of patients.

The IBL is a critical surgical parameter in tumor resections, significantly influencing both postoperative recovery and long-term survival outcomes (36, 37), with substantial evidence identifying it as an independent prognostic factor for tumor recurrence (38) through multiple interconnected pathophysiological mechanisms. First, excessive hemorrhage induces hypovolemia-triggered stress responses that suppress T-cell and NK-cell mediated immune surveillance against residual tumor cells (39). Second, blood loss promotes systemic release of pro-inflammatory cytokines including IL-6 and TNF-*α*, establishing a chronic inflammatory environment conducive to tumor proliferation and metastasis (40). Third, IBL causes tissue hypoperfusion leading to intestinal ischemia–reperfusion injury, with subsequent oxygen free radical generation which induces DNA damage and deterioration of the tumor microenvironment (39). Fourth, IBL increases susceptibility to postoperative complications such as anastomotic leakage, infection, and thromboembolic events, which collectively affect rehabilitation (39, 41). Relevant studies have demonstrated cancer-specific impacts: in CRC, increased IBL elevates complication rates and delays adjuvant therapy, diminishing long-term survival (42); in non-small cell lung cancer cases, greater hemorrhage correlates with disease recurrence and mortality, potentially via immunosuppression and inflammation (43); while gastric cancer studies associate elevated IBL with both increased complications and delays in adjuvant therapy—both of which impair survival (44). Particularly in RC populations, augmented IBL shows strong associations with both local recurrence and distant metastasis (42). Consistent with these findings, our study revealed that IBL > 130 mL independently predicts poorer survival in stage I-III RC patients, whereas those maintaining ≤130 mL demonstrate significant survival advantages. Consequently, clinical implementation of surgical approaches that minimize blood loss and rigorous intraoperative hemostasis protocols is helpful to optimize long-term oncological outcomes.

Despite the widespread clinical application of pTNM staging for prognostic evaluation in RC patients, its predictive accuracy remains limited by the exclusion of critical individual variables, including nutritional status, immune function, dynamic tumor marker fluctuations, and IBL. Thus, this limitation contributes to substantial survival heterogeneity among patients with identical pTNM stages. Contemporary research has sought to address this limitation by developing prognostic models that integrate pTNM staging with patients’ individual characteristics. In esophageal cancer, radiomic features combined with PNI demonstrated predictive capacity for lymph node metastasis (45). However, the extraction and analysis of patients’ radiomic features require professional techniques and equipment, which may restrict the application of the model in some primary medical institutions. Similarly, in patients undergoing radical gastrectomy for gastric cancer, combining PNI with CEA and CA242 demonstrated promising clinical value for the early prediction of postoperative anastomotic leakage (46), yet insufficient external validation compromises its generalizability. Xu et al. identified preoperative CEA/PNI ratio, lymph node metastasis, perineural invasion, surgical approach, and postoperative chemotherapy as independent prognostic factors for CRC patients, and developed a nomogram that demonstrated superior predictive performance compared to individual parameters. However, this model’s robustness may be compromised by the omission of clinically significant covariate, such as IBL, and by limited statistical power due to an undersized validation cohort (47). In contrast, our study is the first to identify pTNM stage, preoperative PNI, preoperative CEA, IBL, and postoperative CEA as independent risk factors for the survival of stage I-III RC patients undergoing surgery. Based on these five factors, we developed a nomogram incorporating the preoperative nutritional index (PNI). Calibration plot analysis showed that the nomogram had high accuracy in predicting the postoperative survival rate of RC patients. Comparative ROC analysis revealed higher AUC values for our PNI-incorporated nomogram relative to both non-PNI-nomogram and pTNM staging models, suggesting a potential improvement in discriminative capacity. Decision curve analysis indicated an incremental clinical net benefit at 3-year timepoints compared to these alternative models. Additionally, the PNI-incorporated nomogram appeared to exhibit higher clinical utility in predicting 3-year OS versus 5-year OS, potentially offering more support for short-term clinical decision-making. Thus, it might hold particular value for medium-term and short-term risk stratification. Notably, all incorporated variables are routinely accessible clinically, which could facilitate its application as a practical tool for individualized postoperative prognosis assessment and therapeutic decision-making in stage I-III RC patients.

However, this study still has several limitations. First, the model excludes patients with preoperative metastases, thereby limiting its applicability for predicting survival outcomes in the metastatic patients. Second, the model excludes psychosocial factors, such as distress, anxiety, and depression, that may affect treatment adherence and physical recovery. Third, the study is a single-center retrospective study and lacks more external validation. Additionally, the model exhibited a decline in predictive performance on the external validation set compared to the training set, suggesting a certain degree of overfitting and indicating that the model’s robustness requires further improvement. Therefore, future studies should incorporate additional relevant risk factors, expand sample sizes, optimize feature engineering, explore more advanced model architectures or ensemble learning methods, and include more diverse validation cohorts to enhance the generalizability of the model. Multicenter studies and prospective clinical trials are also warranted to improve its predictive accuracy and clinical utility.

In conclusion, our research demonstrates that the PNI, an easily accessible and cost-effective biomarker, is a significant prognostic determinant for postoperative survival in stage I-III RC patients with surgical intervention. Furthermore, the improved PNI-incorporated nomogram, which incorporates preoperative pTNM staging, PNI, IBL and CEA levels 6 months after surgery, demonstrates superior predictive accuracy and clinical applicability compared to conventional pTNM staging systems. This tool provides actionable guidance for personalized management of RC patients.

## Data Availability

The raw data supporting the conclusions of this article will be made available by the authors, without undue reservation.
